# Immunotherapy in hepatocellular carcinoma: evaluation and management of adverse events associated with atezolizumab plus bevacizumab

**DOI:** 10.1177/17588359211031141

**Published:** 2021-07-29

**Authors:** Chiun Hsu, Lorenza Rimassa, Hui-Chuan Sun, Arndt Vogel, Ahmed O. Kaseb

**Affiliations:** Graduate Institute of Oncology, National University College of Medicine, National University Hospital, and National University Cancer Center, Taipei; Department of Biomedical Sciences, Humanitas University, Pieve Emanuele, Milan, Italy; IRCCS Humanitas Research Hospital, Humanitas Cancer Center, Medical Oncology and Hematology Unit, Rozzano, Milan, Italy; Department of Liver Surgery and Transplantation, Liver Cancer Institute, Zhongshan Hospital, Fudan University, Shanghai, China; Department of Gastroenterology, Hepatology and Endocrinology, Medical School Hannover, Carl-Neubergstrasse 1, Hannover, 30625, Germany; Gastrointestinal Medical Oncology, The University of Texas MD Anderson Cancer Center, 1515 Holcombe Blvd, Unit 426, Houston, TX 77030, USA

**Keywords:** antiangiogenic agent, bleeding, endoscopy, immune checkpoint inhibitor, portal hypertension, safety

## Abstract

In light of positive efficacy and safety findings from the IMbrave150 trial of atezolizumab plus bevacizumab, this novel combination has become the preferred first-line standard of care for patients with unresectable hepatocellular carcinoma (HCC). Several additional trials are ongoing that combine an immune checkpoint inhibitor with another agent such as a multiple kinase inhibitor or antiangiogenic agent. Therefore, the range of first-line treatment options for unresectable HCC is likely to increase, and healthcare providers need succinct information about the use of such combinations, including their efficacy and key aspects of their safety profiles. Here, we review efficacy and safety data on combination immunotherapies and offer guidance on monitoring and managing adverse events, especially those associated with atezolizumab plus bevacizumab. Because of their underlying liver disease and high likelihood of portal hypertension, patients with unresectable HCC are at particular risk of gastrointestinal bleeding, and this risk may be exacerbated by treatments that include antiangiogenic agents. Healthcare providers also need to be alert to the risks of proteinuria and hypertension, colitis, hepatitis, and reactivation of hepatitis B or C virus infection. They should also be aware of the possibility of rarer but potentially life-threatening adverse events such as pneumonitis and cardiovascular events. Awareness of the risks associated with these therapies and knowledge of adverse event monitoring and management will become increasingly important as the therapeutic range broadens in unresectable HCC.

## Introduction

The standard of care for first-line systemic treatment of unresectable hepatocellular carcinoma (HCC) has, since 2007, been sorafenib^1,2^ or, since 2018, lenvatinib, each of which is an orally administered multi-kinase inhibitor (MKI).^3^ In 2020, a new option was approved for first-line treatment of unresectable HCC: the combination of atezolizumab, an immune checkpoint inhibitor (ICI) that targets programmed death ligand 1 (PD-L1), with bevacizumab, an antiangiogenic agent that targets vascular endothelial growth factor (VEGF).^4^ Thanks to positive safety and efficacy findings from the phase III IMbrave150 trial, this immunotherapy combination is now the preferred first-line standard of care, as recommended in the recently revised guideline from the American Society of Clinical Oncology (ASCO).^4,5^ Nevertheless, the need remains for more effective treatment of HCC in the first line and beyond. Reflecting this need, immunotherapy-based treatment options are increasing across lines.

Two other ICIs, nivolumab^6^ and pembrolizumab,^7^ both of which target programmed cell-death protein 1 (PD-1), are already in use in the second line in the United States, and in April 2021, the Oncologic Drug Advisory Committee of the US Food and Drug Administration (FDA) voted against continuation of the accelerated approval of nivolumab (https://www.targetedonc.com/view/odac-opposes-ongoing-fda-approval-of-nivolumab-for-hcc-in-patients-pretreated-with-sorafenib). In 2020, on the basis of phase II data (CheckMate 040 trial, cohort 4; ClinicalTrials.gov identifier: NCT01658878), the US FDA granted accelerated approval for the combination of nivolumab and ipilimumab for patients with progression on or intolerance of sorafenib.^8^ The combination of pembrolizumab and lenvatinib (phase Ib KEYNOTE-524/Study 116 trial; ClinicalTrials.gov identifier: NCT03006926) has been designated a ‘breakthrough therapy’ for the first-line treatment of HCC not amenable to locoregional treatment, and pembrolizumab plus lenvatinib *versus* lenvatinib alone as first-line treatment of advanced HCC continues to be evaluated in the phase III LEAP-002 trial (ClinicalTrials.gov identifier: NCT03713593).^9,10^ Currently, multiple other regimens based on combinations of kinase inhibitors, anti-VEGF agents, and ICIs are being investigated. Combination regimens that include an ICI and are under investigation in the first-line setting in phase III trials are listed in Table 1.^11,12^ In light of this rapidly expanding treatment landscape, how can healthcare providers optimise therapeutic decisions, and what do they need to know about the safety profiles of the new immunotherapy combinations? How are immunotherapy-related adverse events (AEs) best managed? Here, we review the efficacy and safety of individual components of ICI-based combinations, as monotherapies, and the efficacy and safety of the combinations themselves. With a particular focus on the safety profile of atezolizumab plus bevacizumab, we examine clinical factors to consider before initiating this combination therapy in the first line in patients with unresectable HCC and provide recommendations for monitoring and managing key AEs.

**Table 1.** ICI combinations under investigation in the first-line setting in phase III trials in advanced or unresectable HCC.

Combination *versus* comparator	Mechanism of action	ClinicalTrials.gov ID (trial acronym) Completion date
Atezolizumab + cabozantinib *versus* sorafenib *versus* cabozantinib	Anti-PD-L1 + MKI *versus* MKI *versus* MKI	NCT03755791 (COSMIC-312) 1 Dec 2021
Camrelizumab (SHR-1210) ± FOLFOX4 *versus* placebo	Anti-PD-1 ± chemotherapy	NCT03605706Dec 2021
Durvalumab ± tremelimumab *versus* sorafenib	Anti-PD-L1 ± anti-CTLA4 *versus* MKI	NCT03298451 (HIMALAYA) 30 Apr 2022
Lenvatinib + pembrolizumab *versus* lenvatinib + placebo	MKI + anti-PD-1 *versus* MKI	NCT03713593 (LEAP-002) 13 May 2022
Camrelizumab (SHR-1210) + apatinib *versus*sorafenib	Anti-PD-1 + anti-VEGFR2 *versus*MKI	NCT03764293 Jun 2022
Sintilimab + bevacizumab biosimilar IBI305 *versus* sorafenib	Anti-PD-1 + anti-VEGF *versus* MKI	NCT03794440 (ORIENT-32) Dec 2022
Lenvatinib ± CS1003 *versus*lenvatinib	MKI ± anti-PD-1 *versus* MKI	NCT0419477530 Jun 2023
Nivolumab + ipilimumab *versus* sorafenib or lenvatinib	Anti-PD-1 + anti-CTLA4 *versus* MKI	NCT04039607 (CheckMate 9DW) 30 Sep 2023
IBI310 + sintilimab *versus* sorafenib	anti-CTLA4 + anti-PD-1 *versus* MKI	NCT04720716 1 Dec 2023
HLX10 + HLX04 *versus*sorafenib	Anti-PD-1 + anti-VEGF *versus* MKI	NCT04465734 15 Mar 2024
SCT-I10A + bevacizumab biosimilar SCT-510 *versus* sorafenib	Anti-PD-1 + anti-VEGF *versus* MKI	NCT04560894 Sep 2024
Penpulimab injection + anlotinib *versus* sorafenib	Anti-PD-1 + MKI *versus* MKI	NCT04344158 31 Dec 2024
Toripalimab (JS001) + bevacizumab *versus* sorafenib	Anti-PD-1 + anti-VEGF *versus* MKI	NCT04723004 31 Dec 2024

CTLA4, cytotoxic T-lymphocyte-associated protein 4; HCC, hepatocellular carcinoma; ICI, immune checkpoint inhibitor; MKI, multi-kinase inhibitor; PD-1, programmed cell-death protein 1; PD-L1, programmed death ligand 1; VEGF, vascular endothelial growth factor; VEGFR, vascular endothelial growth factor receptor.

## Components of ICI-based combinations: their efficacy and safety as monotherapy

Efficacy and safety data on MKI monotherapy (sorafenib and lenvatinib in the first line, and regorafenib and cabozantinib in the second line) are summarised elsewhere.^3,13,14^ Here we present known AEs associated with these monotherapies. Common side effects of sorafenib are, briefly, hand–foot skin reaction, diarrhoea, and weight loss.^11^ Patients with more advanced liver disease experience greater numbers of AEs leading to dose reduction or drug discontinuation,^12^ but post-marketing data have shown sorafenib to be tolerable in some patients with Child–Pugh B cirrhosis.^11^ Compared with sorafenib, lenvatinib is associated with higher rates of grade ⩾3 hypertension (23% *versus* 14%), proteinuria (6% *versus* 2%), and anorexia (5% *versus* 1%).^11^ Regorafenib and cabozantinib have similar safety profiles, the most common grade ⩾3 AEs being hand–foot skin reaction, hypertension, diarrhoea, and fatigue.^11^

Bevacizumab, an extensively characterised antiangiogenetic agent thanks to 15 years of use in multiple cancer indications,^15^ has also shown a signal of activity and is well tolerated as monotherapy in patients with advanced HCC.^16,17^ Bleeding is a known safety concern in cancer patients who receive VEGF- or VEGF receptor-targeted therapies^18,19^ and may represent a special concern in HCC patients. For example, in the REFLECT trial, haemorrhagic events of any grade occurred in 23% of patients in the lenvatinib arm and in 15% of patients in the sorafenib arm; grade 3 or 4 bleeding or haemorrhagic events occurred in 4% of patients in each of the lenvatinib and sorafenib arms.^20^ In the SHARP trial of sorafenib *versus* placebo, the incidences of serious haemorrhagic events were 9% *versus* 13%, and of variceal bleeding, 2% *versus* 4%, respectively.^1^

Key efficacy data from studies of ICIs as monotherapy in advanced or unresectable HCC are listed in Table 2(a). Nivolumab and pembrolizumab were each approved by the FDA as second-line therapy for advanced HCC on the basis of phase II data showing that durable objective responses were achieved in 15–20% of patients.^11^ As monotherapy, atezolizumab was evaluated (in comparison with atezolizumab plus bevacizumab) in arm F of the phase 1b GO30140 study [Table 2(b)]. Patients who received atezolizumab alone had a median follow-up of 6.7 months and a median progression-free survival (PFS) of 3.4 months.^21^ Safety data on these agents (and others) as monotherapy are summarised in Table 3(a). In the CheckMate 040 study of nivolumab the most common treatment-emergent AEs (TEAEs) were rash, elevated transaminases, increased amylase and lipase, and pruritus.^6^ TEAEs with pembrolizumab (KEYNOTE-224 study) were similar; immune-mediated AEs occurred in 14% of patients.^7^ In unresectable HCC, there is no study-derived evidence to suggest that one ICI demonstrates an improved efficacy and safety profile over any other.

**Table 2.** Efficacy data from key studies of ICIs as (a) monotherapy or (b) in combination in advanced or unresectable HCC. (a).

ICI	Study type	OS (95% CI), months	PFS (95% CI), months	ORR per RECIST v1.1
Anti-PD-1				
Nivolumab	phase I–II (CheckMate 040, NCT01658878)^6^	15.0 (9.6–20.2)(dose-escalation phase)NR in dose-expansion phase	3.4 (1.6–6.9) (dose-escalation phase) 4.1 (3.7–5.5) (dose-expansion phase)3.7 (3.1–3.9) *versus* 3.8 (3.7–4.5)	15% (dose-escalation phase) 20% (dose-expansion phase)
	phase III (*versus* sorafenib; CheckMate 459, NCT02576509)^22^	16.4 (13.9–18.4) *versus* 14.7 (11.9–17.2) (HR 0.84, *p* = 0.0419). Primary endpoint of OS not met		15% *versus* 7%
Pembrolizumab	phase II (KEYNOTE-224, NCT02702414)^7^phase III (*versus* placebo; KEYNOTE-240, NCT02702401)^23^	12.9 (9.7–15.5)13.9 (11.6–16.0) *versus* 10.6 (8.3–13.5) HR 0.781 (0.611–0.998), *p* = 0.0238	4.8 (3.4–6.6)3.0 (2.8–4.1) *versus* 2.8 (1.6–3.0) HR 0.718 (0.570–0.904),*p* = 0.0022	17%18% *versus* 4% (*p* = 0.00007) DCR 62% *versus* 53% DoR 13.8 months
		Co-primary endpoints (improving OS and PFS) not met		
Camrelizumab^24^	phase II (NCT02989922)^25^phase II^26^	6-month OS: 74.4%(68.0–79.7%)17.0 months in patients with RCCEP *versus* 5.8 months in those without (*p* < 0.0001)	2.1 (2.0–3.2)3.2 months in patients with RCCEP *versus* 1.9 months in those without (*p* < 0.0001)	14.7%19.3% in patients with RCCEP *versus* 5.6% in those without (*p* = 0.0044)
		In multivariable analyses, development of RCCEP was significantly associated with prolonged PFS and OS after adjusting for baseline covariates		
Tislelizumab	phase III (RATIONALE 301, NCT03412773)^27^ (preliminary data)	NA	NA	12.2% (PR in 6 and SD in 19 of 49 patients evaluable for response) DCR 51%Median DoR 15.7 months
Anti-PD-L1				
Atezolizumab	See GO30140 study, below			
Anti-CTLA4				
Tremelimumab	phase II (NCT01008358)^28^phase I–II (with ablation; NCT01853618)^29^	8.2 (4.6–21.3)	6.48 (3.95–9.14)	17.6%; DCR 76.4%
		12.3 (9.3–15.4)	7.4 (4.7–19.4)^a^	Not reported

a Time to progression.

**Table:** (b).

ICI combination	Study type	OS (95% CI), months	PFS (95% CI), months	ORR per RECIST v1.1
Atezolizumab + bevacizumab *versus* atezolizumab	phase Ib (GO30140,NCT02715531)^30^	NA	5.6 (3.6–7.4) *versus* 3.4 (1.9–5.2) (HR 0.55, *p* = 0.0108)	36% DCR 71%
Atezolizumab + bevacizumab *versus* sorafenib	phase III (IMbrave150, NCT03434379)^4^Data cut-off 29 August 2019	12 months:67.2% (61.3–73.1%) *versus* 54.6% (45.2–64.0%)	6.8 (5.7–8.3) *versus* 4.3 (4.0–5.6)HR 0.59, *p* < 0.001	27% *versus* 12%
Atezolizumab + bevacizumab *versus* sorafenib	phase III (IMbrave150, NCT03434379)^31^Data cut-off 31 August 2020	Median 19.2 (17.0–23.7) *versus* 13.4 (11.4–16.9)HR 0.66, *p* = 0.0009	6.9 (5.7–8.6) *versus*4.3 (4.0–5.6)HR 0.65, *p* = 0.001	30% *versus* 11%
Camrelizumab + apatinib	phase I (NCT02942329)^32^	NA	NA	31%
Camrelizumab + apatinib	phase II (RESCUE,NCT03463876)^33^	12 months:74.7% (62.5–83.5%)in first-line cohort68.2% (59.0–75.7%)in second-line cohort	5.7 (5.4–7.4)in first-line cohort5.5 (3.7–5.6)in second-line cohort	34% in first-line cohort 23% in second-line cohort
Durvalumab + tremelimumab	phase I–II (NCT02519348)^34^	T300+D: 18.7 (10.8–NR)T75+D: 11.3 (8.4–14.6)	DoR NR (T300+D) and13.2 months (T75+D)	T300+D: 22.7%T75+D: 9.5%
Sintilimab + bevacizumab biosimilar IBI305 *versus* sorafenib	phase II–III (ORIENT-32, NCT03794440)^35^	NR *versus* 10.4HR 0.569 (0.431–0.751) *p* < 0.0001	4.6 *versus* 2.8HR 0.565 (0.455–0.701)*p* < 0.0001	20.5% *versus* 4.1%

CI, confidence interval; CTLA4, cytotoxic T-lymphocyte-associated protein 4; DCR, disease control rate; DoR, duration of response; HCC, hepatocellular carcinoma; HR, hazard ratio; ICI, immune checkpoint inhibitor; NA, not assessed or not applicable; NCT, ClinicalTrials.gov; NR, not reached; ORR; objective response rate; OS, overall survival; PD-1, programmed cell-death protein 1; PD-L1, programmed death ligand 1; PFS, progression-free survival; PR, partial response; RCCEP, reactive cutaneous capillary endothelial proliferation; SD, stable disease; T300+D, tremelimumab 300 mg + durvalumab 1,500 mg every 4 weeks for 1 dose followed by durvalumab 1,500 mg every 4 weeks; T75+D, tremelimumab 75 mg + durvalumab 1,500 mg every 4 weeks for 4 doses followed by durvalumab 1,500 mg every 4 weeks.

**Table 3.** Safety data from key studies of ICIs as (a) monotherapy or (b) in combination in advanced or unresectable HCC. (a).

ICI	Study type	Treatment-related AEs		Permanent discontinuation because of AEs
		Any grade	Grade ⩾ 3	
Anti-PD-1				
Nivolumab	phase I–II (CheckMate 040, NCT01658878)^6^phase III (*versus* sorafenib;CheckMate 459,NCT02576509)^22^	NA	25% (AST and ALT increase, lipase and amylase increase, pruritus)	11%
		NR	22% *versus* 49%	4% *versus* 8%
Pembrolizumab	phase II (KEYNOTE-224, NCT02702414)^7^	73% (serious in 15%). Immune-mediated hepatitis 3%60.9% *versus* 48.5%. Immune-mediated hypo- or hyperthyroidism, pneumonitis: 18.3% *versus* 8.2%. Immune-mediated hepatitis: *n* = 10 (pembrolizumab group). Steroid use 8.2% *versus* 0.7%	Grade 3: 24% (elevated AST or ALT, fatigue). Grade 4: 1% (hyperbilirubinaemia).1 death (ulcerative oesophagitis)18.6% *versus* 7.5%.Elevated AST 5.4% *versus* 1.5%.Elevated ALT 3.6% *versus* 1.5%.Immune-mediated hypo- or hyperthyroidism, pneumonitis 7.2% *versus* 0.7%	5%
	phase III (*versus* placebo; KEYNOTE-240, NCT02702401)^23^			17% *versus* 9%
Camrelizumab^24^	phase II (NCT02989922)^25^	RCCEP in 66.8%^26^	22%.Elevated AST 5%.Reduced neutrophil count 3%.2 deaths (1, liver dysfunction; 1, multiple organ failure)	4%
Sintilimab	Case report^36^	Autoimmune diabetes	NA	NA
Tislelizumab	phase III (RATIONALE 301, NCT03412773)^27^(preliminary data)	Reduced appetite (*n* = 14), rash (*n* = 12), decreased weight (*n* = 11), cough (*n* = 10)	1 death (acute hepatitis confounded by rapid PD)	NR
Anti-PD-L1				
Atezolizumab	See GO30140 study, below			
Anti-CTLA4				
Tremelimumab	phase II (NCT01008358)^28^(*n* = 20)	Rash 65%, fatigue 55%, diarrhoea 30%, syncope 15%, insomnia 15%, abdominal pain 10%	Rash 5%, diarrhoea 5%, syncope 10%	NR
		No steroids needed because of severe immune-mediated AEs; transient but intense transaminase elevation after first dose only		
	phase I–II (with ablation; NCT01853618)^29^(*n* = 32)	Grade ⩾2.Hyperbilirubinaemia (*n* = 10).AST increase (*n* = 18).ALT increase (*n* = 9).Pruritus (*n* = 4).Rash (n = 5)	Hyperbilirubinaemia (*n* = 3).AST increase (*n* = 7).ALT increase (*n* = 3).Pruritus (*n* = 1).Rash (*n* = 0)	13%
		One patient received steroid treatment for rash		

**Table:** (b).

ICI combination	Study type	Treatment-related AEs		Permanent discontinuation because of AEs
		Any grade	Grade ⩾3	
Atezolizumab + bevacizumab *versus* atezolizumab	phase Ib (GO30140 arm F, NCT02715531)^30^	68% *versus* 41%	20% *versus* 5%	NR
Atezolizumab + bevacizumab *versus* sorafenib	phase III (IMbrave150, NCT03434379)^4^	Hypertension 23.7%.Proteinuria 18.8%.Fatigue 15.2%.AST increase 14.0%.Pruritus 13.1%.Infusion-related reaction 10.9%.Diarrhoea 10.3%.ALT increase 10.3%.Decreased appetite 10.0%	Hypertension 10.3%.Proteinuria 2.7%.Fatigue 1.5%.AST increase 4.3%.Pruritus 0.Infusion-related reaction 2.1%.Diarrhoea 0.3%.ALT increase 2.1%.Decreased appetite 0.6%	15.5% *versus* 10.3%
Camrelizumab + apatinib	phase I (NCT02942329)^32^	NR	60.6%	NR
Camrelizumab + apatinib	phase II (RESCUE)^33^	Serious 28.9%	77.4%.Hypertension 34.2%.2 treatment-related deaths	12.1%
Durvalumab + tremelimumab	phase I–II (NCT02519348)^34^	NR	T300+D: 35.1% T75+D: 24.4% (+ 1 grade 5 AE^a^)	T300+D: 10.8% T75+D: 6.1%
Sintilimab + bevacizumab biosimilar IBI305 *versus* sorafenib	phase II–III (ORIENT-32, NCT03794440)^35^	88.7% *versus* 93.5%	33.7% *versus* 35.7%	13.7% *versus* 5.9%

a Hepatic failure.

AE, adverse event; ALT, alanine aminotransferase; AST, aspartate aminotransferase; CTLA4, cytotoxic T-lymphocyte-associated protein 4; HCC, hepatocellular carcinoma; ICI, immune checkpoint inhibitor; NA, not assessed or not applicable; NCT, ClinicalTrials.gov; NR, not reported; PD, progression of disease; PD-1, programmed cell-death protein 1; PD-L1, programmed death ligand 1; RCCEP, reactive cutaneous capillary endothelial proliferation; T300+D, tremelimumab 300 mg + durvalumab 1,500 mg every 4 weeks for 1 dose followed by durvalumab 1,500 mg every 4 weeks; T75+D, tremelimumab 75 mg + durvalumab 1,500 mg every 4 weeks for 4 doses followed by durvalumab 1,500 mg every 4 weeks.

## Efficacy and safety of ICI-based combinations

Combination immunotherapy strategies for HCC treatment are in ongoing development because only a modest proportion of patients receive a treatment benefit from immune checkpoint inhibition as monotherapy. Immune checkpoint inhibition reactivates the antitumour immune response through blockade of immune exhaustion or inhibitory pathways that are induced by the immune system’s chronic response to tumour antigens. In comparison, the combined approach targets multiple biological mechanisms of action in addition to blockade of immune exhaustion pathways. Those additional mechanisms are (1) release of tumour antigen to prime the tumour antigen-specific T-cell response, (2) increase in the frequency of tumour-specific cytotoxic T cells and their homing to the tumour microenvironment, and (3) tumour microenvironment remodelling strategies such as normalisation of blood supply to remove the hypoxic and immunosuppressive microenvironment.^37^ The effectiveness of this approach has been confirmed by the outcomes of immunotherapy combination trials for the treatment of unresectable HCC; as a result, the European Medicines Agency has now approved one ICI-based combination (atezolizumab plus bevacizumab), while two such combinations are currently approved by the US FDA (nivolumab plus ipilimumab, and atezolizumab plus bevacizumab).

Key efficacy data on immunotherapy combinations, including atezolizumab plus bevacizumab, are listed in Table 2(b). Treatment with three different regimens of nivolumab plus ipilimumab was tested in the CheckMate 040 trial, in which patients in arm A (*n* = 50) received nivolumab 1 mg/kg plus ipilimumab 3 mg/kg every 3 weeks (four doses) followed by nivolumab 240 mg intravenously every 2 weeks; those in arm B (*n* = 49) received nivolumab 3 mg/kg plus ipilimumab 1 mg/kg every 3 weeks (four doses), again followed by nivolumab 240 mg intravenously every 2 weeks; and those in arm C (*n* = 49) received nivolumab 3 mg/kg every 2 weeks plus ipilimumab 1 mg/kg every 6 weeks.^38^ The overall response rate (ORR) was highest in arm A [32%, 95% confidence interval (CI) 20–48%], in which 4 patients had a complete response and 12 a partial response (PR); the median duration of response (DoR) in arm A was 17.5 (4.6–30.5+) months, and 31% of responses lasted ⩾24 months.^38^ Additionally, in the Chinese ORIENT-32 trial of the anti-PD1 antibody sintilimab plus a bevacizumab biosimilar *versus* sorafenib, median overall survival (OS) and PFS were significantly longer and the ORR significantly higher with the immunotherapy combination than with sorafenib at a median follow-up of 10.0 months, and OS and PFS were consistently longer with the combination than with sorafenib across multiple subgroups.^35^ In the IMbrave150 trial, at the 29 August 2019 data cut-off, the median PFS was significantly longer with atezolizumab plus bevacizumab than with sorafenib [6.8 (95% CI 5.7–8.3) months *versus* 4.3 (95% CI 4.0–5.6) months] whereas median OS was not evaluable in the atezolizumab plus bevacizumab arm.^4^ However, at the 31 August 2020 data cut-off, median OS was 19.2 months with atezolizumab plus bevacizumab *versus* 13.4 months with sorafenib [hazard ratio (HR) 0.66 (95% CI 0.52–0.85); *p* = 0.0009], thus showing a consistent, clinically meaningful treatment benefit on the combination therapy.^31^ Notably, given that the majority of HCC patients also have underlying chronic liver disease, future clinical trials should also assess the utility of evaluating the deterioration of liver function as a clinically meaningful, secondary time-to-event endpoint.

Treatment-related AEs and other key safety data on immunotherapy combinations in advanced or unresectable HCC are listed in Table 3(b). In CheckMate 040, the most common treatment-related AEs of any grade with nivolumab plus ipilimumab were (in arm A) pruritus (45%), rash (29%), diarrhoea (24%), aspartate aminotransferase (AST) increase (20%), and hypothyroidism (20%). Patients in arm A had the highest rates of AEs in general and of immune-mediated AEs (grade 3 or 4 hepatitis occurred in 20% of patients); 90% of immune-mediated AEs (including hepatic events) were resolved across treatment arms by using protocol-specified management algorithms.^8,38^ Patients in arm A also had the highest rate of treatment discontinuation due to treatment-related toxic effects (22% *versus* 6% in arm B and 2% in arm C).^38^ In ORIENT-32, rates of treatment-related AEs were 88.7% (any grade) and 33.7% (grade 3 or 4) in the sintilimab plus bevacizumab biosimilar group and 93.5% (any grade) and 35.7% (grade 3 or 4) in the sorafenib group.^35^ The combination was most commonly (in >20% of patients) associated with any-grade proteinuria, reduced platelet count, increased AST, hypertension, and increased blood bilirubin. Treatment was discontinued because of AEs by 13.7% of the combination arm and by 5.9% of the sorafenib arm.^35^ In the IMbrave150 trial, the most common grade 3 or 4 AE with atezolizumab plus bevacizumab was hypertension (15.2%), consistent with the known safety profile of bevacizumab. Grade 5 AEs occurred in 4.6% of the atezolizumab plus bevacizumab group and in 5.8% of the sorafenib group.^4^ Serious AEs were more common with atezolizumab plus bevacizumab (38.0%) than with sorafenib (30.8%), and the incidence of upper gastrointestinal (UGI) bleeding was 7% in the atezolizumab plus bevacizumab group *versus* 4.5% in the sorafenib group.^4^ When interpreting the AE incidence rate, the longer duration of treatment with atezolizumab plus bevacizumab should be taken into account (median duration of treatment: 7.4 months for atezolizumab and 6.9 months for bevacizumab, *versus* 2.8 months for sorafenib). At present, no time-adjusted analysis has been performed. An updated analysis of safety data from the IMbrave150 trial (29 August 2019 data cut-off; median duration of follow-up of 8.6 months) showed that AEs of particular relevance to atezolizumab (hepatitis, rash, hypothyroidism, infusion-related reaction, hyperthyroidism, pancreatitis, and diabetes mellitus) occurred in 68.7% of patients receiving atezolizumab plus bevacizumab and in 82.1% of patients receiving sorafenib. AEs of particular relevance to bevacizumab (the known adverse drug reactions of hypertension, bleeding or haemorrhage, proteinuria, and venous or arterial thromboembolic events) occurred in 57.8% of patients receiving atezolizumab plus bevacizumab and 48.7% of patients receiving sorafenib.^39^

## Factors to consider before initiating first-line atezolizumab plus bevacizumab combination therapy

### Bleeding risk

Patients with cirrhosis are at risk of UGI bleeding of variceal and nonvariceal origin.^40,41^ Nonvariceal bleeding occurs mostly from peptic ulcers, which account for 30–40% of nonvariceal UGI bleeding.^40^ Peptic ulcers are common in patients with cirrhosis, for whom mortality rates are higher than for those without cirrhosis when they bleed, and 15% of patients with cirrhosis die within 6 weeks of nonvariceal UGI bleeding.^40–42^

Variceal bleeding is one of the most severe and immediately life-threatening complications and causes 70% of all UGI bleeding events in patients with portal hypertension.^43,44^ Clinically significant portal hypertension is associated with a high risk of gastro-oesophageal varices, which are present in 42% of patients with Child–Pugh A and 72% of patients with Child–Pugh B or C liver function,^43^ but portal hypertension can also be due to treatment or to portal vein invasion (common in patients with HCC). In a recent Korean study that compared patients with and without HCC, patients with HCC had higher rates of 5-day treatment failure, 6-week mortality, and cirrhosis-related complications of acute variceal bleeding.^45^ Model for End-Stage Liver Disease (MELD) score and Barcelona Clinic Liver Cancer (BCLC) stage of HCC were strong predictors of 6-week mortality (85% of patients with a MELD score ⩾15.5 and BCLC stage C or D HCC died within 6 weeks of an acute variceal bleed, and the 6-week mortality risk was 21 times as high in these patients as in those with lower MELD scores and BCLC stage A or B HCC; *p* < 0.001).^45^

Although the presence of untreated or incompletely treated varices and bleeding or high risk of bleeding were exclusion criteria of the IMbrave150 trial, 26% of patients in each treatment arm had varices at baseline. To participate in the trial, patients were required to undergo an oesophago-gastroduodenoscopy (OGD) within 6 months before initiation of therapy: all sizes of varices had to be assessed and active varices treated per local standard of care before study treatment started. The rate of bleeding or haemorrhage of any grade was 25% in the atezolizumab plus bevacizumab arm and 17% in the sorafenib arm.^4^ In the atezolizumab plus bevacizumab arm (*n* = 329), gastrointestinal (GI) haemorrhage occurred in eight patients (and was grade 3 or 4 in four patients); oesophageal varices haemorrhage occurred in eight patients (grade 3 or 4 in six patients), and UGI haemorrhage occurred in four patients (grade 3 or 4 in two patients).^4^ The corresponding numbers in the sorafenib arm (*n* = 156) were, for GI haemorrhage, three patients (all grade 3 or 4); oesophageal varices haemorrhage, one patient (grade 3 or 4); and UGI haemorrhage, two patients (both grade 3 or 4).^4^ The type of bleeding most often associated with atezolizumab plus bevacizumab was epistaxis (grade 1 or 2, in 13% of patients).^21^ We strongly recommend that in clinical practice, patients with cirrhosis (particularly those with portal hypertension) should undergo upper endoscopy to assess their bleeding risk before starting treatment with bevacizumab. Furthermore, annual upper endoscopy should be considered, according to clinical judgement, to re-evaluate varices found at baseline.

Prospective studies have consistently demonstrated that the risk of variceal bleeding, estimated overall at 5–15% per year, is related to the size of the varices.^43,46^ However, this risk is amplified by the presence of red wale marks (places where the variceal wall is thin and therefore weakened) on endoscopy,^43,46^ and ‘high-risk’ varices are not only medium or large (i.e. those that do not collapse with insufflation at endoscopy) but also small, with red signs.^43^ Patients who are very likely to have high-risk varices are those with decompensated cirrhosis, a platelet count ⩽150,000/mm^3^, and liver stiffness ⩾20 kPa (determined by transient elastography),^47,48^ but transient elastography to measure liver stiffness is not routinely available in many parts of the world, including most of those with a high prevalence of liver cirrhosis and HCC. The most widely available tool for assessing bleeding risk is UGI endoscopy. Therefore, we recommend UGI endoscopy to evaluate the risk of bleeding from varices in all patients; UGI endoscopy may also help evaluate bleeding risk in patients with peptic ulcers. We strongly recommend that UGI endoscopy be performed within 6 months before treatment initiation. However, endoscopy-based assessment of bleeding risk can be subjective, and we recommend asking the endoscopist to provide a statement regarding the risk of bleeding associated with varices.

Although in some guidelines endoscopy is not considered necessary in patients with non-alcoholic steatohepatitis or hepatitis B virus (HBV)-related HCC and no cirrhosis, we strongly recommend endoscopy to evaluate the risk of both variceal and nonvariceal bleeding before use of atezolizumab plus bevacizumab in patients in whom portal hypertension and cirrhosis raise a high clinical suspicion of varices. Medium-to-large varices should be treated according to the local standard of care, including a nonselective beta-blocker or endoscopic band ligation to prevent a first episode of variceal bleeding.^48^ We recommend that treatment with atezolizumab plus bevacizumab should not be initiated within 14 days after ligation, because the bleeding risk is highest within the first 10 days.^49^ We also advise repeat endoscopy to re-evaluate the status of varices and bands. Prevention of acute variceal bleeding in patients with HCC should follow the same principles as those for patients without HCC,^47^ and nonselective beta-blocker therapy should also be given to patients with clinically significant portal hypertension.^48^ Management of other risk factors such as peptic ulcer should follow the same principles as those for patients without HCC. Upon diagnosis by upper OGD, peptic ulcers should be treated with a proton pump inhibitor for 6–8 weeks.^50^ In patients with advanced HCC, portal vein thrombosis due to tumour invasion is a major concern, whereas thrombosis due to hypercoagulation is uncommon. It remains unclear whether the risk of portal vein thrombosis is increased by the use of bevacizumab, which has a known association with thromboembolic events.^15^ The use of anticoagulants should be balanced against the bleeding risk associated with bevacizumab.

### Proteinuria and hypertension

VEGF inhibitors, including bevacizumab and ramucirumab, are associated with nephrotoxicity, most commonly proteinuria and hypertension.^51^ Multiple studies have shown that angiogenesis inhibitors induce proteinuria in a dose-dependent manner.^52^ A meta-analysis of seven randomised, controlled trials showed the incidence of bevacizumab-associated proteinuria to range from 21% to 62%; the greatest risk was associated with higher-dose therapy (relative risk 1.4 with low-dose, 2.2 with high-dose bevacizumab).^53^ A study of predictive factors showed that regardless of cancer type (colon, gastric, lung, or breast cancer) or antiangiogenic agent used, the incidence and severity of proteinuria increase with the number of cycles of antiangiogenic agent administered, especially if ⩾13 cycles.^52^ Before initiating therapy with atezolizumab plus bevacizumab, bear in mind that patients with comorbidities such as diabetes or kidney disease may have proteinuria at baseline. Although additional information is needed on the association of proteinuria with accumulated exposure to bevacizumab in patients with HCC, all patients due to receive VEGF inhibition therapy should undergo a thorough assessment of renal function before initiation, including evaluation of serum creatinine.^51^

The meta-analysis mentioned above showed that patients with cancer who received bevacizumab also had a significant and dose-dependent increase in risk of hypertension (relative risk 3.0 with low-dose, 7.5 with high-dose bevacizumab).^53^ Hypertension must therefore be well controlled before starting therapy with atezolizumab plus bevacizumab; the most commonly used treatments are angiotensin-converting enzyme inhibitors and angiotensin II receptor blockers.^54^

### Other factors to consider

Most patients are already receiving treatments that cause diarrhoea, which should be identified and treated. In patients with other cancers, GI AEs are more likely with regimens containing an anti- cytotoxic T-lymphocyte-associated protein 4 (CTLA4) agent and less likely with anti-PD-1 or -PD-L1 monotherapy.^55,56^

A poor Eastern Cooperative Oncology Group performance status (ECOG PS) may be an indicator of an aggressive tumour or poor nutritional status. Patients with a poor ECOG PS (i.e. a score >1) are therefore not candidates for treatment of any kind.

## Monitoring and management of AEs associated with atezolizumab plus bevacizumab combination therapy

Management of immune-related AEs should follow the guidelines of ASCO,^57^ the European Society of Medical Oncology (ESMO),^58^ and the National Comprehensive Cancer Network (NCCN).^59^ Although assessing the occurrence, type, timing, and severity of immune-related AEs is a challenge,^60^ the time course of occurrence of immune-related AEs should be considered. An analysis of time to onset of first AEs of particular relevance with atezolizumab and bevacizumab in the IMbrave150 study showed that the earliest atezolizumab-defined AEs were infusion-related reaction (onset at median 0.7 months), pancreatitis (1.4 months), and hepatitis (1.6 months); rash and hyperthyroidism occurred at a median of 2.7 months, hypothyroidism at 3.5 months, and diabetes mellitus at 4.4 months.^39^ The earliest bevacizumab-defined AE was hypertension (onset at median 1.5 months), followed by proteinuria and venous thromboembolic events at 2.8 months, bleeding or haemorrhage at 3.3 months, and arterial thromboembolic events at 4.7 months.^39^ The onset of GI symptoms is most commonly 5–10 weeks after initiation of immune checkpoint inhibition.^57^

### Bleeding

During treatment, patients should be monitored carefully for bleeding events, which can be life threatening regardless of the type of therapy (atezolizumab plus bevacizumab or an MKI). Bleeding may be monitored by using non-endoscopic methods (haemogram, occult blood if the patient is anaemic, or a decrease in haemoglobin), but for patients with cirrhosis who present with GI bleeding, endoscopy should be performed within 12 h,^48^ and variceal bleeding should be distinguished from nonvariceal bleeding.

In patients with cirrhosis, acute GI bleeding due to varices or nonvariceal lesions is a medical emergency.^43^ Treatment of acute variceal bleeding should follow the same principles in patients without and with HCC; nonselective beta-blockers combined with endoscopic band ligation are recommended for secondary prophylaxis of variceal bleeding.^43,47^ Urgent endoscopic variceal or band ligation is recommended, but it precludes the use of antiangiogenic agents (e.g. bevacizumab) and MKIs (e.g. sorafenib), and atezolizumab monotherapy should be considered, at the clinician’s discretion. The decision to resume treatment with bevacizumab should be taken with great care and based on the results of variceal therapy, the patient’s clinical stability, and multidisciplinary discussion of the risk–benefit ratio.

### Liver function impairment, hepatitis, and viral reactivation

Because most patients have underlying liver disease, a high level of attention to the liver is crucial. An increase in liver transaminases is common in patients receiving immunotherapy, and a rapid rise from baseline in bilirubin, alanine aminotransferase (ALT), or AST serves as an alert and dictates the next treatment steps. We recommend testing ALT, AST, and bilirubin before each treatment cycle.

Liver function impairment (defined by Child–Pugh score) must be distinguished from hepatitis (defined by transaminase elevation). In Child–Pugh A patients, good and impaired liver function are more precisely distinguished by albumin-bilirubin (ALBI) grade.^61^ In patients with Child–Pugh B status at baseline because of cirrhosis and portal hypertension, systemic treatment should be used with caution and with frequent monitoring, given the risk of further liver deterioration on therapy. On the basis of ORR and DoR in CheckMate 040 nivolumab, as a single agent, was granted accelerated approval by the US FDA for the treatment of HCC in patients who have previously received sorafenib: that study population included patients with Child–Pugh A5 (68%), Child–Pugh A6 (31%), and Child–Pugh B7 (1%) liver function.^62^ Although nivolumab has shown promising efficacy and tolerability in HCC patients with Child–Pugh B liver function,^63^ few data are available on the efficacy and safety of ICI therapy in these patients, who are generally excluded from phase III trials and for whom, therefore, no ICIs are fully approved. Patients with Child–Pugh B liver function are a challenging population in which to perform clinical trials. For example, the trial of ramucirumab as second-line therapy for advanced HCC initially enrolled patients with compensated Child–Pugh B cirrhosis, but enrolment was terminated early because of safety concerns.^64^ Therefore, active therapy for patients with Child–Pugh B status should be subject to multidisciplinary discussion and assessment of risk–benefit ratio. We suggest that treatment be considered in fit patients (ECOG PS 0 or 1) whose main disease manifestation is impaired liver function. A patient with mild ascites and normal bilirubin may be a better candidate than one with no ascites but high bilirubin. Enrolment in clinical trials, whenever available, is a priority for this population, to allow prospective monitoring of safety and treatment efficacy.

Multidisciplinary management is necessary to identify the cause (treatment *versus* disease progression *versus* underlying liver disease) of any alteration in liver function test, and care teams should pay particular attention to patients at high risk of decompensation. In case of immune-related hepatitis, immune checkpoint blockade should be temporarily or permanently discontinued and immunosuppressive therapy with corticosteroids administered immediately.^65,66^ Steroid use should be monitored closely, however,^67^ given that it could have detrimental effects, in terms of infection risk and hepatic decompensation, in patients with cirrhosis.^68^ Therefore, diagnosis of immune-related hepatitis should be carefully made before subjecting patients to any risk of steroid use.^69^ Note that diverse factors can lead to hepatitis or increased transaminases during treatment with ICIs, therefore histological examination of liver biopsy tissue may help differential diagnosis, including that of immune-related hepatitis. However, current recommendations are generally based on small cohorts of patients receiving different types of ICIs.^70–72^

For HCC patients with virus-related cirrhosis, a relevant issue is the risk of HBV or hepatitis C virus (HCV) reactivation during systemic anticancer therapy. The IMbrave150 trial enrolled patients with HBV-related HCC only if the HBV infection was well controlled (HBV DNA <500 IU/ml); analysis of the viral kinetics of HBV and HCV and of liver-related AEs suggests that there is not an increased risk of viral reactivation or hepatitis flare with atezolizumab plus bevacizumab, compared with sorafenib, in patients with HBV- or HCV-related HCC.^73^ In the HBV analysis population, the incidence of HBV reactivation was lower in the atezolizumab plus bevacizumab arm than in the sorafenib arm, and HBV flare was not observed in either arm. In the HCV analysis population, incidences of HCV reactivation and flare were comparable in the two treatment arms. In both treatment arms, there were no hepatic AEs or liver failure in patients who had HBV or HCV reactivation, and there was no treatment discontinuation due to HBV or HCV reactivation.^73^ Findings from a case series indicate that, regardless of the initial HBV viral load, ICI therapy may be safely given to patients with HBV-related HCC as long as it is accompanied by antiviral therapy.^74^

### Diarrhoea and colitis

One of the most common serious, immune-related AEs in clinical trials is due to GI tract toxicity; GI AEs are also the most common reason for discontinuation of immunotherapy.^58,67^ Diarrhoea arising during immunotherapy should be evaluated to rule out immune-related colitis, which requires treatment and, if severe (grade ⩾3), hospitalisation. Immune-related colitis manifests as an increase in stool frequency, diarrhoea or constipation, blood or mucus in stool, abdominal pain or cramping, and nausea and vomiting.^55^ Computed tomography (CT) findings of immune-related colitis include mesenteric vessel engorgement, bowel wall thickening, and fluid-filled colonic distention; positron emission tomography and CT scans show diffuse colonic wall thickening.^57^

First-line treatment of diarrhoea (grade ⩾2 or with apparent colitis symptoms), if the stool infectious work-up is negative, is systemic steroids (1–2 mg/kg corticosteroid).^57,59,75^ In the IMbrave150 trial, colitis requiring systemic corticosteroid treatment within 30 days of its onset occurred in 1.2% of patients in the atezolizumab plus bevacizumab arm.^39^ Note that steroid-associated immunosuppression may compromise the antitumour response,^67^ and a long duration of steroid treatment is associated with a higher risk of infections: early nonsteroid immunosuppressive therapy may ensure a more favourable overall outcome.^75^ If symptoms do not improve after 2–5 days of corticosteroid treatment, a stronger immunosuppressive agent (e.g. the tumour necrosis factor (TNF)-α blocker infliximab or the anti-integrin α4β7 antibody vedolizumab) may be effective.^57,59,75^ Immunotherapy should be withheld if immune-related colitis is moderate (grade 2); if grade 3, anti-CTLA4 agents should be discontinued and anti-PD-1 or -PD-L1 agents perhaps resumed after resolution of toxicity, and if grade 4, the responsible agent should be permanently discontinued.^59^ Increased use of immunotherapy combinations may complicate the management of diarrhoea, and multidisciplinary management or expert referral may be necessary.

### Proteinuria

All patients receiving VEGF inhibition therapy (e.g. bevacizumab or ramucirumab) should undergo close monitoring of their renal function (including serum creatinine).^51^ Proteinuria associated with antiangiogenic agents can be a reason to interrupt treatment with bevacizumab. In trials, bevacizumab therapy is withheld for one or two cycles when 24-h urinary excretion exceeds 2 g, but in clinical practice, 24-h urinary excretion is difficult to measure, and dipstick testing generally suffices.

### Other less common but clinically important AEs

Pneumonitis occurs in 3–10% of patients taking checkpoint inhibitors, and this complication is lethal in 0.2–2% of patients.^76^ The most common symptoms are dyspnoea (53%), cough (35%), fever (12%), and chest pain (7%). ICIs should be temporarily discontinued and methylprednisolone 1–2 mg/kg given daily, with antibiotic if indicated.^76^ If this treatment fails, provide additional immune suppression with infliximab, mycophenolate mofetil, or cyclophosphamide. In most cases, checkpoint inhibition can be resumed.

Cardiovascular AEs are rare, occurring in <0.1% of patients receiving ICIs, but they can be life threatening and have been reported with all approved agents.^57^ Attention is particularly necessary in patients with underlying cardiovascular disease. For the initial evaluation of patients with potential cardiovascular toxicity, ASCO recommends electrocardiography, testing for troponin and brain natriuretic peptide, and chest X-ray;^57^ NCCN recommendations include testing for cardiac biomarkers (creatine kinase and troponin) and inflammatory biomarkers (erythrocyte sedimentation rate, C-reactive protein level, and white blood cell count).^59^ ASCO recommends discontinuing immunotherapy if any cardiovascular AE occurs,^57^ whereas the NCCN recommends discontinuation if cardiovascular AEs are severe or life threatening (grade 3 or 4).^59^ Treatment should follow the NCCN, ESMO, or ASCO guidelines.

Some patients are more sensitive than others to elevation of blood pressure during treatment with bevacizumab and therefore need more aggressive antihypertensive control; most other patients need only adjustment of their antihypertensive medication regimen. If necessary, obtain the expertise of a cardiologist to maintain proper control of blood pressure during bevacizumab therapy.

Fatigue can be detrimental to patients’ quality of life and, therefore, treatment adherence: monitor by asking the patient. A clear recommendation to address fatigue is difficult, but exercise may help. Regardless of the type of treatment, if fatigue worsens during therapy investigate the possibility of endocrinopathy through thyroid function tests (T_3_, T_4_, thyroid-stimulating hormone).

## Discussion and conclusions

Thanks to its significant and clinically meaningful efficacy and manageable safety profile, atezolizumab plus bevacizumab has become the preferred first-line therapy of choice for patients with unresectable HCC, thus validating the strategy of combining immunotherapies with other immunotherapy or targeted agents in HCC. With this change in the first-line standard of care, and the anticipated addition of multiple new combination therapies to the treatment armamentarium, comes the need to identify those patients most likely to benefit from any new therapy and to understand its safety and AE management profile. Indeed, a recently reported novel measure of net health benefit,^77^ the incremental safety-effectiveness ratio (ISER),^78^ could help identify patients who may benefit from and tolerate new systemic therapies, thus avoiding harm. Use of such a measure is particularly important in patients with advanced HCC, given the high prevalence of concomitant chronic liver disease in these patients and the associated risk of harm from treatments. The ISER could be a useful secondary endpoint in clinical trials.

Discussion of the efficacy of ICIs and their combinations has focused on classical endpoints such as median OS and PFS, ORR, and DoR. However, immune checkpoint inhibition can have a delayed effect and may be associated with a prolonged DoR, therefore its benefit may not be properly captured by these endpoints. Therefore, while the use of surrogate endpoints in patients with advanced HCC is controversial,^79^ we believe a better assessment of efficacy may be provided by the proportion of patients alive or free of progression at late time points or a restricted mean survival time.^80^ For patients with a substantial tumour burden, ORR and time to response are important.

Consideration of the efficacy of ICIs suggests that immunotherapy is enhanced by VEGF inhibition through an immunomodulatory role in the tumour microenvironment,^81,82^ but it is not clear whether antiangiogenic agents in general have synergistic or additive antitumour effects when combined with anti-PD-1 or anti-PD-L1 therapy. The combination of lenvatinib and pembrolizumab resulted in an ORR of 37.7% by mRECIST per investigator and RECIST 1.1 per independent imaging review (50.0% by mRECIST per independent imaging review) in patients with unresectable HCC,^9^ whereas pembrolizumab as monotherapy results in an ORR < 20% [Table 2(a)], and ORRs with lenvatinib as monotherapy range from only 18.8% (based on independent imaging review per RECIST 1.1) to 40.6% (based on independent imaging review per mRECIST).^20^ These data strongly suggest antitumour synergy between the two drugs. In arm F of the GO30140 study, median PFS was longer in patients who received atezolizumab plus bevacizumab (5.6 months) than in those who received atezolizumab alone (3.4 months);^21^ evidence of antitumour synergy between these two agents may be provided by the long-term outcome of the crossover of 26 of the 59 subjects randomised to atezolizumab monotherapy to the combination therapy arm. Evidence of the efficacy of combinations other than atezolizumab plus bevacizumab (IMbrave150 trial) is limited; available data (from phase I and II trials) suggest promise of an ICI with a kinase inhibitor (pembrolizumab with lenvatinib^9^ or camrelizumab with apatinib^33^). Preclinical studies have indicated that MKIs may exert immunomodulatory effects in the HCC microenvironment independently of their antiangiogenic effects.^83,84^ Although additional immunomodulatory mechanisms may enhance antitumour immunity, the optimum biological effective dose of MKIs should be better defined to avoid treatment-related AEs.^81,85^

Before initiating atezolizumab plus bevacizumab combination therapy, clinicians should consider several important risks in relation to an individual patient’s characteristics. For example, patients with unresectable HCC often have cirrhosis or portal hypertension (or both) associated with oesophageal or gastric varices and portal gastropathy; therefore, the disease alone can increase risk of bleeding, which in turn may be exacerbated by treatment. Indeed, the trial protocol necessitated upper endoscopy within 6 months from study entry to assess (clinical discretion or investigator’s judgement) risk of bleeding. In the IMbrave150 trial, bevacizumab was given at 15 mg/kg every 3 weeks, a dosage at the upper limit of the dosage range used in other cancers. In previous phase II studies in HCC, different doses and schedules of bevacizumab (5 mg/kg and 10 mg/kg every 2 weeks) showed evidence of activity, but this finding has not been validated in the phase III randomised trial setting.^17,86–88^ Although previous studies have suggested a dose-dependent effect of bevacizumab on AEs such as hypertension and bleeding, there is currently no evidence to suggest a correlation between bevacizumab dosage and efficacy.^19,89^ The 15 mg/kg dose of bevacizumab used in the IMbrave150 trial may offer the best possible efficacy, but it can also lead to life-threatening AEs such as bleeding. Clinicians should also be alert to the risk of proteinuria or hypertension, particularly in patients with diabetes or kidney disease, and be aware that the risk of GI AEs such as diarrhoea or colitis is greater with anti-CTLA4 agents than with anti-PD-1 or -PD-L1 agents. That response to anti-PD-1 immunotherapy is affected by the gut microbiome in patients with HCC is of interest.^90^ Finally, clinicians should also remember that a poor ECOG PS precludes therapy of any kind.

Whenever possible, immune-related AEs should be managed according to the most recent ESMO,^58^ ASCO,^57^ or NCCN^59^ guidelines. Awareness of the time course of occurrence of immune-related AEs may aid monitoring. Vigilance is crucial when monitoring for bleeding events, liver function impairment, hepatitis, reactivation of HBV or HCV infection, diarrhoea and colitis, and proteinuria; clinicians should also be alert for rarer AEs such as pneumonitis and cardiovascular events and should maintain control of hypertension, with the help of a cardiologist if necessary. Fatigue may signal endocrinopathy, which should be investigated, and patients with fatigue may need help to adhere to their therapy.

As the range of available therapies increases, immunotherapies may also find use in the neoadjuvant setting (before curative resection or ablation therapy) and in the adjuvant setting (to help reduce the incidence of HCC recurrence after surgical resection or ablation therapy).^91^ With its increased objective response rate and acceptable safety profile, neoadjuvant immunotherapy may potentially improve resectability and treatment outcome in patients with high-risk features for recurrence, such as multiple tumours or macro- or microvascular tumour invasion. The feasibility of using immunotherapy as a bridging therapy for patients who are candidates for liver transplantation, on the other hand, remains unclear. For solid-organ transplant recipients who receive anti-PD-1 or anti-PD-L1 therapy the risk of graft rejection may approach 40%;^92^ in patients who received ICI therapy before transplantation limited data are available.

Several ICIs are under investigation in the adjuvant setting, after curative resection or ablation of HCC. As monotherapy, nivolumab is being investigated in the CheckMate 9DX trial (ClinicalTrials.gov identifier: NCT03383458) in patients who are at high risk of recurrence,^91^ and pembrolizumab is under investigation in the phase III KEYNOTE-937 trial (ClinicalTrials.gov identifier: NCT03867084). Two combinations are being assessed in patients who are at high risk of recurrence: durvalumab with or without bevacizumab in the phase III EMERALD-2 trial (ClinicalTrials.gov identifier: NCT03847428), and atezolizumab plus bevacizumab (*versus* active surveillance) in the phase III IMbrave050 trial (ClinicalTrials.gov identifier: NCT04102098).

Whether ICIs and their combinations are applied in the systemic treatment setting or as neoadjuvant or adjuvant therapy, awareness of therapeutic safety profiles and AE management strategies in patients who receive these drugs is increasingly important. Understanding the safety and AE management profiles of ICI-based combinations such as atezolizumab plus bevacizumab will support appropriate clinical decision-making as the therapeutic range expands in unresectable HCC.
