# Erratum to “Immunotherapy in hepatocellular carcinoma: evaluation and management of adverse events associated with atezolizumab plus bevacizumab”

**DOI:** 10.1177/17588359211047708

**Published:** 2021-09-11

Hsu C, Rimassa L, Sun H-C, *et al.* Immunotherapy in hepatocellular carcinoma: evaluation and management of adverse events associated with atezolizumab plus bevacizumab. *Ther Adv Med Oncol* 2021; 12: 1-20. DOI: 10.1177/17588359211031141

While processing this paper, the affiliation for the author Chiun Hsu was listed incorrectly. The correct affiliation is as given below. This has been corrected in the online version of the article.

Graduate Institute of Oncology, National Taiwan University College of Medicine National Taiwan University Hospital, and National Taiwan University Cancer Center, Taipei, Taiwan

